# Holistic services for people with advanced disease and chronic breathlessness: a systematic review and meta-analysis

**DOI:** 10.1136/thoraxjnl-2018-211589

**Published:** 2018-11-29

**Authors:** Lisa Jane Brighton, Sophie Miller, Morag Farquhar, Sara Booth, Deokhee Yi, Wei Gao, Sabrina Bajwah, William D-C Man, Irene J Higginson, Matthew Maddocks

**Affiliations:** 1 Cicely Saunders Institute of Palliative Care, Policy and Rehabilitation, King’s College London, London, UK; 2 School of Health Sciences, University of East Anglia, Norwich, UK; 3 Department of Oncology, University of Cambridge, Cambridge, UK; 4 NIHR Respiratory Biomedical Research Unit, Royal Brompton and Harefield NHS Foundation Trust and Imperial College, Harefield, UK; 5 Harefield Pulmonary Rehabilitation Unit, Harefield Hospital, Harefield, UK

**Keywords:** palliative care

## Abstract

**Background:**

Breathlessness is a common, distressing symptom in people with advanced disease and a marker of deterioration. Holistic services that draw on integrated palliative care have been developed for this group. This systematic review aimed to examine the outcomes, experiences and therapeutic components of these services.

**Methods:**

Systematic review searching nine databases to June 2017 for experimental, qualitative and observational studies. Eligibility and quality were independently assessed by two authors. Data on service models, health and cost outcomes were synthesised, using meta-analyses as indicated. Data on recipient experiences were synthesised thematically and integrated at the level of interpretation and reporting.

**Results:**

From 3239 records identified, 37 articles were included representing 18 different services. Most services enrolled people with thoracic cancer, involved palliative care staff and comprised 4–6 contacts over 4–6 weeks. Commonly used interventions included breathing techniques, psychological support and relaxation techniques. Meta-analyses demonstrated reductions in Numeric Rating Scale distress due to breathlessness (n=324; mean difference (MD) −2.30, 95% CI −4.43 to −0.16, p=0.03) and Hospital Anxiety and Depression Scale (HADS) depression scores (n=408, MD −1.67, 95% CI −2.52 to −0.81, p<0.001) favouring the intervention. Statistically non-significant effects were observed for Chronic Respiratory Questionnaire (CRQ) mastery (n=259, MD 0.23, 95% CI −0.10 to 0.55, p=0.17) and HADS anxiety scores (n=552, MD −1.59, 95% CI −3.22 to 0.05, p=0.06). Patients and carers valued tailored education, self-management interventions and expert staff providing person-centred, dignified care. However, there was no observable effect on health status or quality of life, and mixed evidence around physical function.

**Conclusion:**

Holistic services for chronic breathlessness can reduce distress in patients with advanced disease and may improve psychological outcomes of anxiety and depression. Therapeutic components of these services should be shared and integrated into clinical practice.

**Registration number:**

CRD42017057508.

**Key messages**

**What is the key question?**

- What are the outcomes, recipients’ experiences and therapeutic components of holistic services for chronic breathlessness in people with advanced disease?

**What is the bottom line?**

- Overall these services reduce patient distress due to breathlessness and may improve psychological outcomes of anxiety and depression.
- Despite wide variability in content and delivery, recipients value tailored interventions and expert staff providing person-centred, dignified care.

**Why read on?**

- This is the first review to synthesise available quantitative and qualitative evidence around holistic services triggered by breathlessness, which may serve as an appropriate referral indicator for early integration of palliative care.

## Introduction

Breathlessness is a common and distressing symptom of chronic disease, affecting almost all people with chronic respiratory disease,^1^ the majority with heart disease or cancer,^1^ and significant proportions of those with renal disease, neurological conditions or HIV/AIDS.^1 2^ With our ageing population and increasing multimorbidity,^3^ the number of people affected by breathlessness worldwide will rise. Breathlessness increases as disease progresses^4^ and often becomes chronic (ie, it persists despite optimal treatment of the underlying disease^5^). The symptom can result in fear, sleep disturbance, social isolation and disability for patients and carers.^6 7^ Breathlessness also occurs alongside other troublesome symptoms such as cough, fatigue and anxiety, and serves as a marker of overall symptom burden and deterioration.^8 9^

There are limited pharmacological treatments for breathlessness: moderate evidence supports use of low-dose opioids,^10 11^ and there is little to support use of benzodiazepines.^12^ As these approaches do not address the psychosocial problems that underlie and perpetuate the symptom,^13^ non-pharmacological treatments take priority. In many people, breathlessness is successfully managed via rehabilitation services that incorporate exercise training, education and behaviour change interventions.^14 15^ Pulmonary rehabilitation, for example, improves functional status and quality of life, and is a cornerstone of best standard breathlessness care.^14^ However, issues with referral, uptake and completion limit reach, particularly to people with the most advanced disease with high levels of disability.^6 16 17^

Holistic services are emerging, designed specifically for those with advanced disease and chronic breathlessness.^18–21^ These typically draw on palliative care, but with integrated working from multiple specialties and professional groups. Treatments are selected based on the physical, psychological, social and spiritual needs of individual patients, and their families or carers. Individual studies suggest a positive impact on health outcomes.^18–21^ For example, an integrated palliative and respiratory care service improved breathlessness mastery, and suggested a potential survival advantage.^21^ Recent international guidelines subsequently advocate for early integration of palliative care in people experiencing chronic disease,^22 23^ and refractory and/or distressing breathlessness may serve as an appropriate referral indicator, especially in non-cancer conditions where prognostication causes delays.^24^ However, the evidence base to guide practice and policy is poorly understood.

We therefore aimed to synthesise available evidence around holistic breathlessness services for people with advanced disease. Our objectives were to describe structures and therapeutic components; determine clinical and cost-effectiveness; and understand patients’ and carers’ experiences of these services.

## Methods

### Design and registration

This systematic review and meta-analysis was conducted and reported in accordance with the Preferred Reporting Items for Systematic Reviews and Meta-Analyses (PRISMA) statement^25^; the protocol was prospectively registered (PROSPERO: CRD42017057508).^26^

### Inclusion and exclusion criteria

#### Participants

Adults experiencing breathlessness related to advanced disease, including cancer (advanced local or metastatic), chronic respiratory disease (GOLD stage III–IV/grade C–D), heart failure (New York Heart Association stage III–IV) or progressive neurological conditions. Studies were eligible if ≥50% of participants met these definitions.

#### Interventions and comparators

In the absence of a standard definition, we defined holistic breathlessness services as those where patients are enrolled due to their breathlessness (not their diagnosis); drawing on skills from multiple specialties and disciplines; using a holistic approach encompassing non-pharmacological and pharmacological interventions as indicated; and supporting self-management. Interventions were excluded if they did not specifically target patients with breathlessness; or used single treatments (eg, breathing training alone). Pulmonary rehabilitation and disease-specific services (eg, integrated respiratory care) were deemed outside the scope of this review. Exclusively targeted service provider or carer interventions were excluded. All comparators were considered.

#### Outcomes

Health outcomes included breathlessness intensity, affect and impact domains^27^; anxiety and depression; physical function; health status or quality of life; and survival. Cost outcomes of interest included service costs and utilisation, and quality-adjusted life-years (QALYs) derived from generic quality of life measures (eg, EuroQol-5D). Experience outcomes included patient and carer perspectives.

#### Designs

Randomised controlled trials (RCTs) and non-RCTs, observational studies and qualitative studies were included. Narrative reviews, opinion papers and case series with <5 participants were excluded. Our search strategy is shown in Box 1.

Box 1 **Search strategy**

**Electronic searches**

The following electronic databases were searched from their inception up to 2 June 2017:

- British Nursing Index
- CINAHL
- Cochrane Database of Systematic Reviews
- Central Register of Controlled Trials
- Database of Abstracts of Reviews of Effectiveness
- EMBASE
- MEDLINE
- PsycINFO
- Science Citation Index Expanded

Search terms were informed by literature scoping and information specialists, and piloted to ensure inclusivity. Subject headings and free text terms were combined to search for population and intervention terms (online supplementary appendix 1 shows the MEDLINE strategy).

**Handsearching**

Reference lists of retrieved studies and relevant reviews, citations, textbooks and voluntary sector materials were searched, and we contacted active researchers for unpublished data/grey literature. No language or publication status restrictions were imposed.

**Screening**

Records were imported into Endnote X7^56^ and duplicates removed. Two authors (SM, LB/MM) screened titles and abstracts for relevance, and independently assessed full texts of potentially eligible studies against eligibility criteria. Disagreements were resolved by discussion, and consultation with a third author (IH) to reach consensus.

10.1136/thoraxjnl-2018-211589.supp1 Supplementary data

### Quality assessment

Two authors (LB, MM) independently assessed the quality of included studies using the Standard Quality Assessment Criteria for Evaluating Primary Research Papers (QualSyst),^28^ which contains checklists for quantitative and qualitative studies. For mixed-method studies, both checklists were used. QualSyst scores are summarised as a percentage score of applicable items. Information to aid quality assessment was obtained from primary, secondary and protocol articles. For RCTs we also assessed risk of bias using the Cochrane Collaboration tool.^29^

### Data extraction and analysis

Data were extracted by one author (SM/LB) using a predesigned electronic form and checked by a second author to ensure rigour (LB/MM). Data were extracted on service characteristics (staff; contacts; duration; interventions; target population), study information (country; authors; year; design) and outcomes. Where additional information was needed for inclusion in meta-analysis, authors were contacted. For experience data, all text (including quotations) under the headings of ‘results’ or ‘findings’ in qualitative or mixed-methods studies were imported verbatim into qualitative data software (NVIVO V.12).^30^

Service characteristics and details of associated studies were tabulated. Component interventions were tabulated and summarised narratively. Data from controlled studies were included to estimate effectiveness. Outcomes were analysed as continuous data where possible. Mean differences (MDs) between intervention and comparator groups were reported with 95% CIs. Where data permitted, meta-analysis was performed using random-effects models, and heterogeneity assessed using the I^2^ 2 statistic. In all cases, individual studies were only represented once within each analysis. Sensitivity analyses excluded studies with high risk of bias (<70% QualSyst score) and removed outliers where substantial heterogeneity (I^2^ >75%)^31^ was present. We planned funnel plots to assess reporting bias if ≥10 studies were included.^32^ Additional findings were summarised narratively.

Qualitative data were coded line by line, and descriptive themes were developed encompassing the themes or codes of primary studies. From these, new analytical themes going beyond presentation of primary data were generated.^33^ Particular attention was paid to similarities and differences across studies, and divergent cases. Multiple stakeholders (researchers, patient/carer representatives, clinicians involved in delivery of services) reviewed the analysis and interpretation to ensure comprehensiveness and increase validity. Data were integrated at the level of interpretation and reporting.

## Results

**Figure 1:**
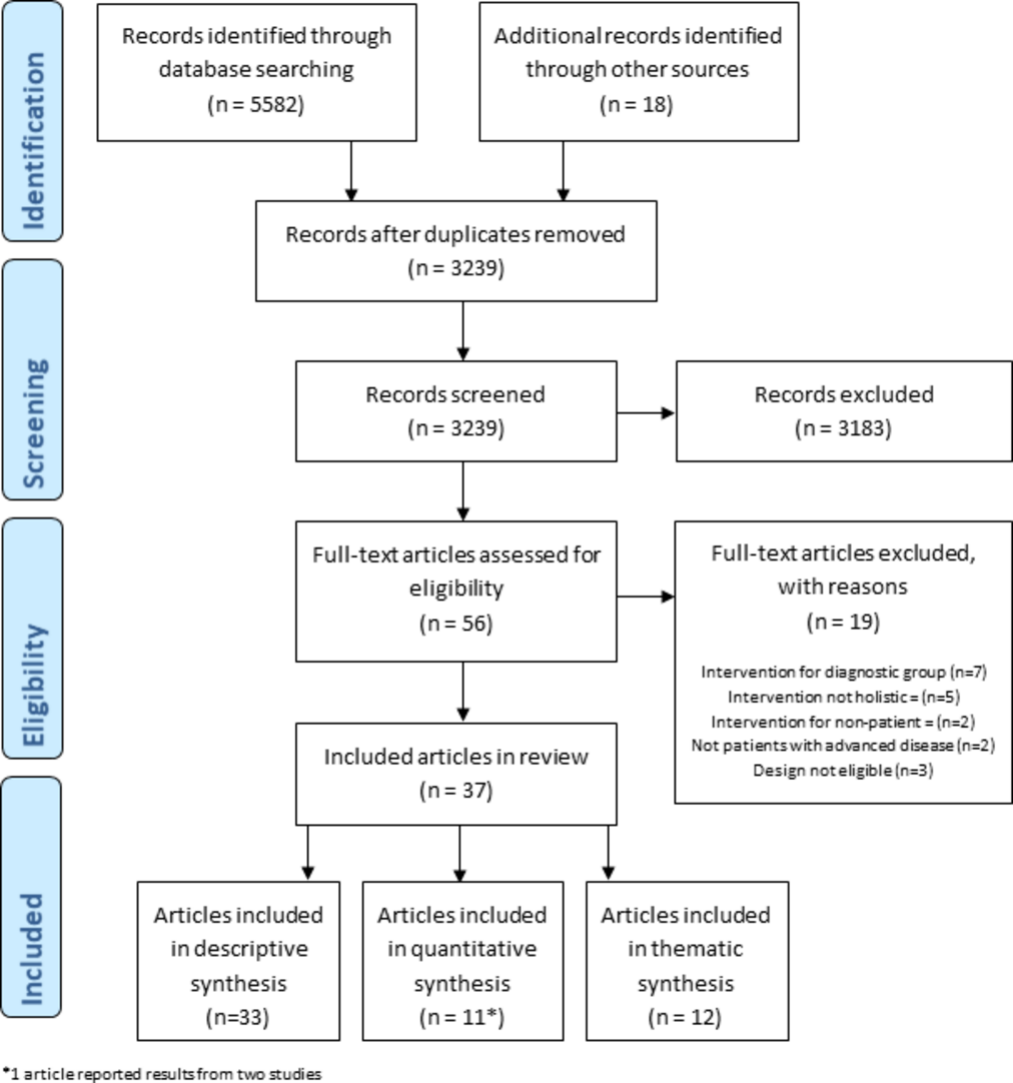
Preferred Reporting Items for Systematic Reviews and Meta-Analyses flow diagram.

Of 3239 unique records identified and 56 full texts screened, 37 articles were eligible for inclusion (figure 1). Articles were published in the period 1996–2017 (27 since 2010) and related to 18 separate holistic breathlessness services: 12 based in the UK, 3 in Canada and 1 each in Australia, Germany and Hong Kong.

### Service characteristics

Thirty-three articles were included in the descriptive synthesis (tables 1 and 2). Most of the services (12 of 18) were delivered to people with advanced cancer and used a mixture of face-to-face and phone contacts (median 4–6, range 1–12) and were short term, usually over 4–6 weeks (range 1–12; table 1). Service providers included doctors, nurses, physiotherapists and occupational therapists, with involvement from palliative care, respiratory care and oncology. Services used a wide range of interventions (table 2), most commonly breathing techniques, psychological support and relaxation or calming techniques. A minority (≤2 of 18) included acupressure/transcutaneous electrical nerve stimulation, sleep hygiene advice, spiritual support or smoking cessation interventions.

**Table 1:** Description of included services and studies

Service description						Studies included in the quantitative and thematic synthesis					
Service	Country	Conditions	Discipline/staff	Contacts	Duration (weeks)	Author (year)	Design	Effectiveness data		Experience data	
								N	Quality (%)	N	Quality (%)
Ahmadi et al* 57	Canada	Lung cancer	Nursing Occupational therapy Social work Palliative care (doctors) Oncology (doctors)	2–3 face-to-face 1 class Phone contact for opioid follow-up if needed	4–6						
Chan *et al* 58	Hong Kong	Cancer	Occupational therapy Physiotherapy Home-care nurses Palliative care (doctors, nurses)	*Inpatients:* daily face-to-face, then two post-discharge *Home-care*: three face-to-face, then six weekly if needed 1+phone calls	4						
Connors *et al* 48 59	UK	Intrathoracic malignancy	Palliative and respiratory (physiotherapist)	5 face-to-face	1–8	Wood *et al* (2013)^48^	Qualitative study	–	-	9	*85*
Corner *et al* 18 34 60	UK	Lung cancer or mesothelioma	Trained nurse research practitioners working alongside respiratory clinics	3–8 clinic visits 3–4 phone calls	8–12	Corner (1996)^18^	Mixed-methods RCT (pilot)	20	*88*	20	*60*
						Bredin (1999)^34^	RCT	102	*81*	–	*–*
Douglas et al* 61	UK	Cancer and non-cancer	Respiratory physiotherapist	1–3 clinic visits	1–4						
Farquhar *et al* 19 20 41–43 62–65	UK	Cancer and non-cancer	Occupational therapy Physiotherapy Palliative care (doctor) Access to: respiratory medicine, psychologist	2–4 home visits 3–4 phone calls	4–8	Booth (2006)^41^	Qualitative study	**–**	*–*	19	*85*
						Farquhar (2010a)^42^*	Qualitative study	**–**	*–*	Missing	*40*
						Farquhar (2010b)^43^	Mixed methods before-after study (pilot)	**–**	*–*	13	*45*
						Farquhar (2014)^19^	Mixed-methods RCT	54	*100*	20	*85*
						Farquhar (2016)^20^	Mixed-methods RCT	79	*100*	20	*80*
Goffin et al* 66	Canada	Intrathoracic malignancy	Oncology (doctor) Palliative care (doctor) Respiratory (therapist, doctor) Nursing	1 clinic visit, follow-up needed							
Hately *et al* 46	UK	Lung cancer or mesothelioma	Clinic run by specialist palliative care physiotherapist	3 clinic visits	4–6	Hately *et al* (2003)^46^	Uncontrolled mixed-method study	–	*–*	30	*50*
Higginson *et al* 21 44 45 47 67 68	UK	Cancer and non-cancer	Physiotherapy Occupational therapy Palliative care (nurse, social worker, doctor) Respiratory care (doctor)	2 clinic visits 1 home visit 3–4 phone calls	6	Higginson *et al* (2014)^21^	Mixed methods RCT	82	*100*	20	*70*
						Gysels *et al* (2015, 2016)^44 45^	Qualitative	–	*–*	20	*80*
						Reilly *et al* (2016)^47^	Cross-sectional postal survey	–	*–*	25	*70*
Johnson *et al* 39 40	UK	Lung cancer	Varied by site; could include Physiotherapy Occupational therapy Oncology (nurse) Palliative care	1 face-to-face versus 3 face-to-face Both with one phone call	2–4	Barton *et al* (2010)^39^	Feasibility RCT	22	*92*	–	*–*
						Johnson *et al* (2015)^40^	RCT	124	*92*	–	*–*
Kachuik et al* 69	Canada	Lung cancer	Physician Nurse Occupational therapist Respiratory therapist Social worker Above with oncology and palliative care expertise	Clinic visits as needed							
McMahon et al* 70	Ireland	Idiopathic pulmonary fibrosis or COPD	Advanced nurse practitioner led Physiotherapist Occupational therapist		4–6						
Pearce et al* 35	UK	COPD	COPD nurse Physiotherapy Occupational therapy	4 clinic visits	4	Pearce *et al* (2006)^35^*	RCT	51	*54*	–	*–*
Schunk *et al* 49 71	Germany	Cancer and non-cancer	Palliative care consultants Respiratory physicians Physiotherapists Access to psychologists, social workers and nurses	2 clinic visits 4 home visits Phone calls as needed	6						
Scullion *et al* 72	UK	Lung cancer	Oncology (nurse) Physiotherapy Occupational therapy Dietician	4 group sessions	4						
Ung et al* 73	Canada	Lung cancer	Multidisciplinary team, including a ‘clinical champion’, tailored by local services.	Precise methodology left to individual cancer centres							
Yates et al* 36 37	Australia	Lung cancer	Nurse led Based on Corner’s integrated model of dyspnoea	4 face-to-face or phone	4	Yates *et al* * (2007)^37^	Quasi-experimental (pilot) and RCT (pilot)	30 and 57	*35–46*	–	*–*
						Yates *et al* * (2011)^36^	RCT	144	*69*	–	*–*
Yorke *et al* 38	UK	Lung cancer	Specialist nurses Physiotherapists Complementary therapists	2 face-to-face 1 phone call	4	Yorke *et al* (2015)^38^	RCT (feasibility)	71	*92*	–	*–*

*Abstract only.

COPD, chronic obstructive pulmonary disease; RCT, randomised  controlled trial; UK United Kingdom.

Quality assessments were completed for studies providing data to the quantitative or thematic synthesis.

**Table 2:** Service components

Intervention	n	Services*
Information and education		
Education/advice	9	18 20 21 38 49 57 58 69 73
Nutritional advice/support	3	20 58 72
Sleep hygiene	2	20 66
Smoking cessation advice/support	1	20
Written information	4	20 21 49 58
Psychosocial support		
Carer/family support	5	18 20 21 49 73
Psychological support	12	18 20 21 35 37 46 49 58 59 69 72 73
Social support	7	18 20 21 35 37 49 58
Spiritual support	1	21
Self-management strategies		
Breathing techniques	14	18 20 21 35 37 38 40 46 49 57–59 66 70
Emergency/crisis planning	3	20 21 49
Exercise plans	5	20 21 49 58 61
Handheld fan/water spray	5	20 21 49 58 59
Goal-setting	4	18 20 49 59
Pacing	8	20 21 40 46 49 58 59 72
Positioning	4	20 21 49 58
Relaxation/calming techniques	11	18 20 21 35 40 46 49 58 59 70 72
Other interventions		
Accupressure/TENS	2	38 61
Occupational aids	5	20 21 49 58 61
Pharmacological review	4	20 21 66 70

*One citation per service.

TENS, transcutaneous electrical nerve stimulation.

### Effectiveness of holistic breathlessness services

Twelve studies (11 RCTs^18–21 34–40^ and 1 quasi-experimental design^37^) from seven services were included in the quantitative synthesis (table 1). Of these five were designed as pilot/feasibility studies^18 36 38 39^ and seven as effectiveness studies.^19–21 34–36 40^ Nine studies compared the services to usual care; in one study^18^ the control group were encouraged to talk freely about their breathlessness and disease but not offered training or counselling, and two studies compared one versus three contacts with a service.^39 40^

Nine studies enrolled only patients with cancer,^18 19 34 36–40^ two enrolled only patients with non-malignant disease^20^ or COPD^35^ and one study enrolled patients with any advanced disease.^21^ In total, 979 patients were recruited (range 22^39^ to 156^40^), including 757 (77.3%) with advanced cancer and 180 (18.4%) with advanced COPD. The remaining participants (4.3%) had other non-malignant diseases including interstitial lung disease or heart failure. A wide variety of outcomes were measured (online supplementary table S1). The most common measures were breathlessness intensity (10 studies), distress due to breathlessness (10 studies) and anxiety and depression (9 studies). Breathlessness intensity measures varied by type (average/best/worst), context (at rest/on exertion) and timing (current/past 24 hours/past week).

**Figure 2:**
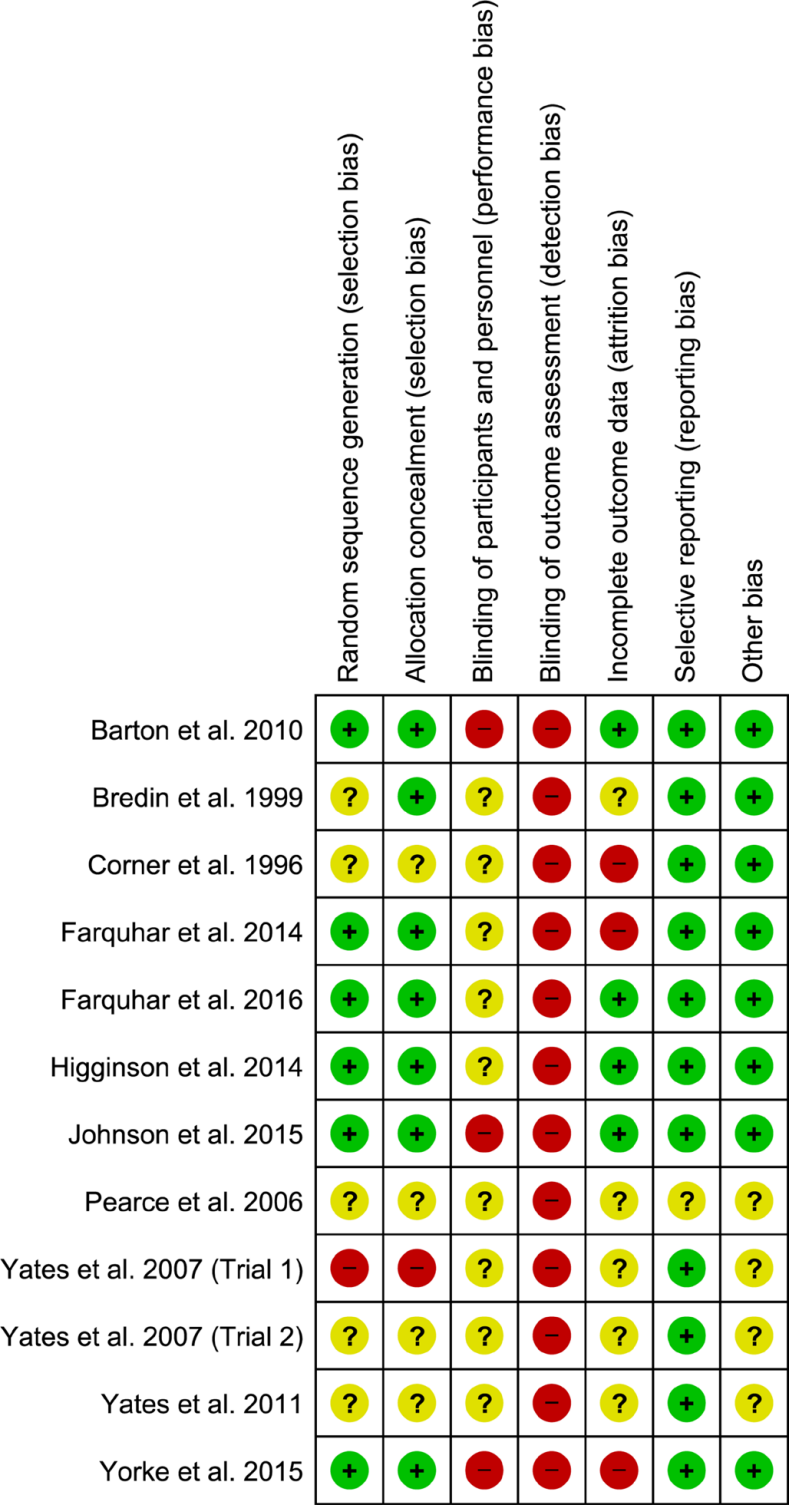
Risk of bias summary.

Quality assessment scores ranged from 35% to 100% (median 90.4%; online supplementary table S2). The lowest scores were for studies where only an abstract was available.^35–37^ Due to the nature of the intervention that prohibits patient blinding and prioritises self-assessed outcomes, all studies were deemed at risk of detection bias and most at risk of performance bias (figure 2). Only three studies reported blinding of investigators.^19–21^

#### Breathlessness intensity

Ten studies^18 21 34–40^ assessed the severity of breathlessness using one or more of the following measures: visual analogue scale (VAS), Numeric Rating Scale (NRS) or Borg scores (see online supplementary table S3 for details). For ‘best breathlessness’ two studies using VAS found a greater improvement compared with control (differences in median change 5.7, p=0.03^34^ and 1.0, p=0.02)^18^ and three studies with unspecified measures found a significant intervention effect (F[2,44]=5.30, p=0.009)^37^ or no difference (data not reported).^36 37^ For ‘worst breathlessness’ one study using VAS found a greater improvement compared with control (difference in median change 3.5, p=0.05),^18^ whereas no significant differences were found by two studies using NRS (MD –0.35, 95% CI –1.71 to 1.01, p=0.61^21^; MD 0.41, 95% CI −0.86 to 1.67, p=0.53),^38^ one study using VAS (difference in median change 3.8, p=0.14)^34^ and one with an unspecified measure (data not reported).^36^ For ‘average breathlessness’ one study using an unspecified measure found a greater improvement compared with control (difference in mean change 1.2,^36^ whereas two studies using NRS did not (MD −0.33, 95% CI −1.28 to 0.62, p=0.49^21^; MD 0.65, 95% CI −0.49 to 1.80, p=0.26).^38^ One study using NRS found no effect on breathlessness on exertion (MD −0.73 95% CI −1.69 to 0.22, p=0.13),^21^ and one study using Borg scale ratings for breathlessness at rest and on exertion found no difference between groups (data not reported).^35^ In line with their feasibility study results,^39^ a powered trial comparing one with three service contacts found no significant difference in NRS worst (MD 0.2, 95% CI −2.31 to 2.97, p=0.83) or average (MD 0.3, 95% CI −2.00 to 2.62, p=0.79) breathlessness.^40^

#### Breathlessness affect

**Figure 3:**
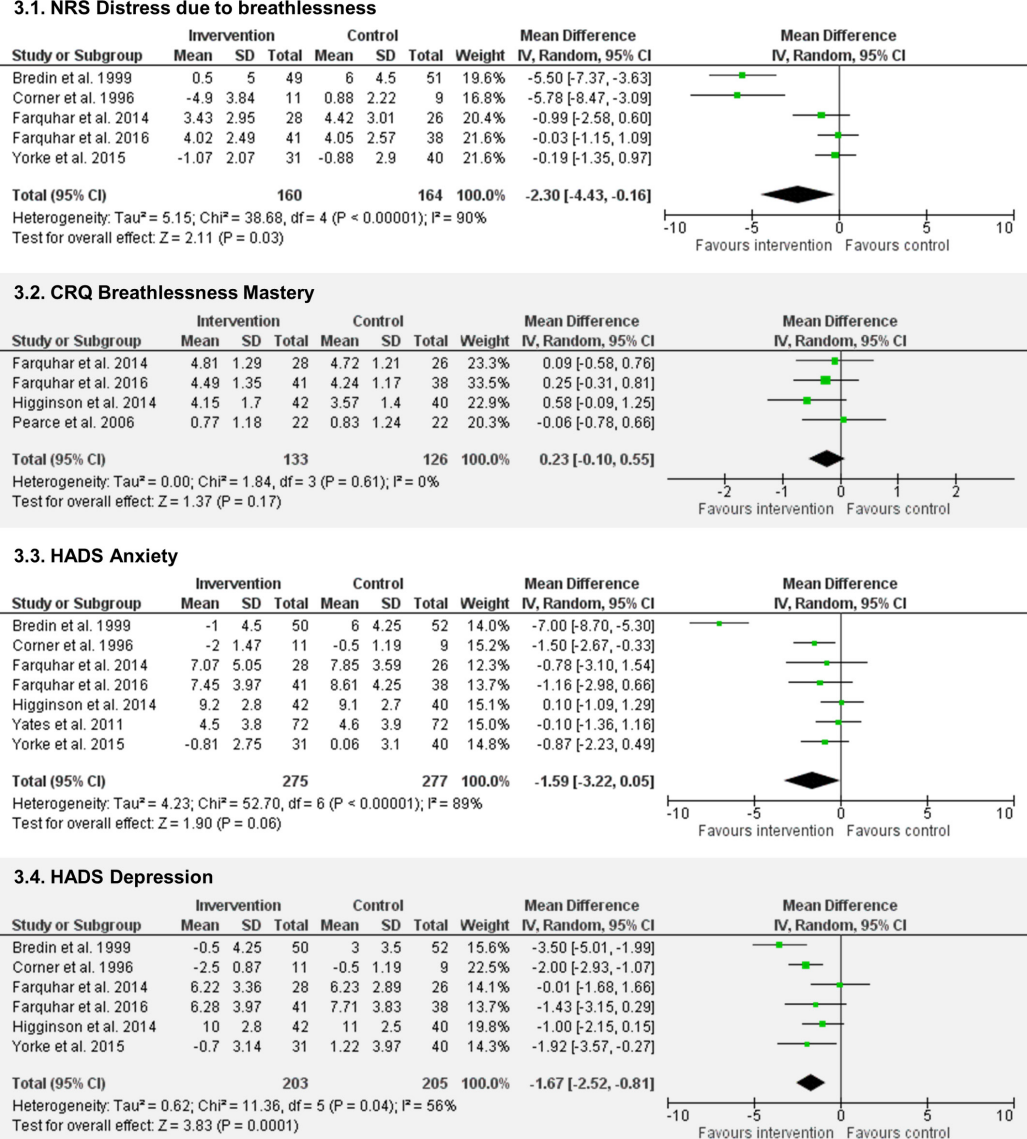
Meta-analyses. HADS, Hospital Anxiety and Depression Scale; NRS, Numeric Rating Scale.

Ten studies^18–20 34 36–40^ assessed ‘distress due to breathlessness’ using VAS (range 0–100, higher=worse) or NRS (range 0–10, higher=worse), two as a prespecified primary outcome.^19 20^ Of eight studies comparing breathlessness services to usual care, data from five^18–20 34 38^ were pooled in a meta-analysis (n=324, figure 3A). Three studies^36 37^ reported no significant difference but could not be included as data were not reported. Meta-analysis of those studies reporting data showed significantly lower NRS distress following the intervention compared with control (MD −2.30, 95% CI −4.43 to −0.16, p=0.03). A sensitivity analysis excluding two outlier studies^18 34^ resulted in a reduced point estimate of effect and non-significant difference (MD −0.29, 95% CI −1.00 to 0.43, p=0.43; I^2^=0%). One feasibility study^39^ and one randomised trial^40^ testing service variations found no difference on NRS ‘coping with breathlessness’ (MD −1.7, 95% CI −4.27 to 0.90, p=0.20)^40^ and significantly higher NRS distress due to breathlessness following three sessions versus one session (MD 3.9, 95% CI 0.98 to 6.91, p=0.01).^40^

Four studies^19–21 35^ assessed ‘mastery over breathlessness’ using the Chronic Respiratory Questionnaire mastery domain; two as a primary outcome.^21 35^ A meta-analysis of these data (n=259, figure 3B) showed a statistically non-significant increase in mastery (range 1–7, higher=better) favouring the intervention (MD 0.23, 95% CI −0.10 to 0.55, p=0.17). A sensitivity analysis excluding one study^35^ deemed at high risk of bias increased the point estimate of effect (MD 0.30, 95% CI −0.06 to 0.66, p=0.11). One study found significantly lower mastery scores following three compared with one service contact (MD −0.6, 95% CI −1.06 to −0.11, p=0.02).^40^

One further study found improved dyspnoea-12 (range 0–36; higher=worse) scores following intervention as compared with control (MD 5.19, 95% CI 0.62 to 9.75, p=0.026).^38^

#### Psychological outcomes

Seven studies assessed anxiety and depression using the Hospital Anxiety and Depression Scale (HADS).^18–21 34 36 38^ Data from these seven studies (n=552, figure 3C) showed a statistically non-significant reduction in anxiety scores (range 0–21; higher=worse) favouring the intervention (MD −1.59, 95% CI −3.22 to 0.05, p=0.06). Sensitivity analysis excluding one study^36^ deemed at high risk of bias increased the point estimate (−1.85, 95% CI −3.76 to 0.06, p=0.06). Sensitivity analysis removing one outlier study^34^ resulted in a reduced point estimate but statistically significant group difference (MD −0.66,–1.23 to −0.10, p=0.02; I^2^=0%). No statistical differences in anxiety were reported when comparing one and three contacts.^39 40^

For depression, one study^37^ reporting no difference between groups could not be included in the meta-analysis as data were not provided. Meta-analysis using six remaining studies (n=408, figure 3D) showed reduced depression scores (range 0–21, higher=worse) favouring the intervention (MD −1.67, 95% CI −2.52 to −0.81, p<0.001). No statistical differences in depression were reported when comparing one and three contacts.^39 40^

Three further studies reported no significant differences between the intervention and control groups in ‘psychological symptoms’; two using an unspecified measure (data not reported)^36 37^ and one using the Rotterdam Symptom Checklist (range 7–28, higher=worse; difference in median change −8, p=0.21).^34^ One study comparing one session with three sessions found no significant difference on CRQ emotion scores (MD −0.09, 95% CI −0.54 to 0.36, p=0.69).^40^

#### Physical function, health status and survival

Five studies^18–21 34^ assessed physical function. Two studies found greater improvements following intervention compared with control using the Functional Capacity Scale (range 0–14; higher=better; MD for change 1.25, p<0.02)^18^ and WHO Performance Scale (range 0–5, higher=worse; difference in median change −2, p=0.02),^34^ respectively. Three studies observed no difference in functional outcomes between groups assessed using either the London Chest Activities of Daily Living Scale^21^ (MD –5, 95% CI –12.22 to 1.02, p=0.10) or patient-reported number of times out of house^19 20^ (data not reported).

Seven studies^19–21 34 35 38 40^ included a measure of health status or quality of life. No significant differences were found between groups across the CRQ dyspnoea domain^19–21 35^ (including the comparison between one and three sessions^40^) or total score,^21^ EuroQol-5D index^21^ or VAS,^21 38^ and the Rotterdam Symptom Scale quality-of-life domain.^34^ Due to heterogenous measures, change from baseline and post-intervention scores, and cases of non-normally distributed data, we decided against meta-analysis for these outcomes.

Two studies reported survival data.^21 34^ One found a significant difference in survival (generalised Wilcoxon score 3.9, p=0.048) in favour of the intervention.^21^ Subgroup analysis found the difference was driven by participants with non-cancer diagnoses. The remaining study, enrolling only patients with cancer, found no difference in survival across groups (data not reported).^34^

### Economic evaluation

One service did not increase formal care costs compared with usual care (mean (SD) £2911 (£2729) vs £3709 (£4484); incremental QALY gain 0.092 (95% CI −0.23 to 0.04)).^21^ Another service^19 20^ was more cost-effective than usual care for patients with cancer (total costs £354 lower (95% CI  – £1020 to £246); incremental QALY-gain 0.0002 (95% CI −0.001 to 0.002)),^19^ but not non-cancer conditions (total costs £799 higher (95% CI  – £237 to £1904); 0.003 QALY gain (95% CI –0.001 to 0.007)).^20^ A third service enrolling patients with cancer found a non-significant reduction in QALYs following three sessions compared with one session (MD −0.006 (95% CIs −0.018 to 0.006)).^40^

### Experiences of holistic breathlessness services

Twelve articles^18–21 41–48^ reporting experience data from five separate services were included in the qualitative synthesis (table 1). These included six mixed-method^18–21 43 46 47^ and five qualitative studies.^41 42 44 45 48^ Most data were from patient and/or carer interviews^18–21 41–45 48^; one used therapists notes,^46^ and one used free-text responses to a postal survey.^47^ Data represented views of 167 patients (53.9% with cancer) and up to 49 carers. Quality assessment scores ranged from 40% to 85% (median 70%; online supplementary table S4). Common limitations included lack of reflexivity, not using verification procedures to establish credibility and unclear reporting of the analytic methods.

Three themes were identified: valued characteristics, perceived outcomes and challenges to services.

Patients valued the education and information sharing included in the services, particularly to help them understand their breathlessness, legitimise the treatments being suggested, and provide resources to refer to in future crises (Box 2). The treatments themselves (breathing techniques, pacing, positioning, relaxation, handheld fan) were praised for their simplicity, portability and perceived effectiveness. The psychosocial support received through the services was highly valued, providing opportunities for participants to have their experiences listened to and acknowledged, receive support and reassurance, and discuss problems beyond their breathlessness. Participants appreciated when carers were involved, both to support them as individuals and in caring for the patient. Overwhelmingly, recipients commented on the qualities of the staff providing services, whom they deemed experts in not only managing breathlessness, but in person-centred care and treating participants with respect and dignity.

Box 2 **Example quotes for themes derived from qualitative synthesis**

**Valued service characteristics**

- Education and information sharing: *'*When I’m having problems I go back and read it to see if I am doing the right thing. I find that very, very helpful.’ (Man, ILD)^21^
- Caring and expert service providers: ‘Would you like a cup a tea […] it’s just human to human situation. But that environment makes you: you are in the right place, you know. There is no guessing going on, […]. You are gonna get the best of their mind.’ (P01043, man with COPD)^45^
- Involving carers: ’Knowing now that he won’t die in one of these sort of situations, so that certainly helped me, and it certainly helped me to realise that, you know, I can probably help him to calm down. So yes, as a carer I think it was a help.’ (038t3c)^20^
- Psychological support: ‘I was able to discuss my personal feelings, that you don’t talk to your family about so not to worry them.’ (69-year-old woman with COPD)^47^
- Simple, portable and effective tools: ’To put my hand on my tummy … puff puff puff … and do that, and you know, it’s amazing really, it sounds so pathetic when you say something … It is simple, it’s not a thing you’d think of doing.’ (530t3pc)^19^

**Perceived outcomes**

Affective distress

- Increased self-efficacy: ‘They were increasing activity and functional levels by using breathing techniques and exploiting the confidence these gave them.’ (Researcher comments)^18^
- Feeling more ‘in control’: ’The blissful thing is, like I’ve said is, you can control your breathing, if you get a bad spell you can work your way through it.’ (Man, ILD)^21^
- Feeling less isolated: ‘At this time you’re down and lonely anyway so having someone there for support is important.’ (04M)^48^
- Increased understanding: ’I was thrilled to bits to be able to be getting some knowledge of what my complaint was all about … that they’re doing something about it.’ (P04)^41^
- Reduced distress: ’Learning to relax and not get so stressed out, I mean I still get the old panic now and again when I’ve really put myself out […], but most of the time I deal with it quite easily.’ (Patient with cancer)^44^

Sensory-perceptual experience

- Perceived impact on what breathing feels like: ‘It helped me to learn to relax, learn to breathe in a more controlled way.’ (54-year-old man with COPD)^47^

Symptom impact or burden

- Improved daily activities: ’Went up and down [the stairs] with very little increase in respiratory rate. Flung her arms around my neck and said "I never thought I would be able to do that again".’ (Staff comment 11)^46^
- Increased self-management: ’I would get into a panic when I was breathless, but now I can sit down use my fan, wet my face, read my laminate (breathlessness poem) and I calm down.’ (Woman with COPD)^21^

**Challenges to services**

- Motivation: ’She gave me a fan and told me to, you know, put it on … and then blow out. I do try to do it, but I get so out of breath doing it. I give up.’ (Case 013)^43^
- Expectations: ’Hoping that something would help me but a little bit cynical as well … I didn’t see how anything could help improve it.’ (03M)^48^

In line with quantitative findings, perceived outcomes were mostly psychological, including increased understanding and self-efficacy, and feeling more ‘in control’, less isolated, or less distressed (Box 2). Some recipients also reported their breathing feeling easier and more controlled. Others felt the sensation of breathlessness was unchanged, but their reaction to it had changed. Participants reported being more able to maintain and/or increase their daily activity, and successfully self-manage.

Two potential challenges for services were identified (Box 2). First, respondents’ accounts showed the importance of motivation to self-manage in the success of the interventions, yet this was difficult if benefits were not quickly achieved. Second, some participants had low expectations of the services or the treatments offered, which at times resulted in a reluctance to engage.

## Discussion

This review synthesised quantitative and qualitative evidence to provide a detailed understanding of holistic services for people with advanced disease and breathlessness that persists despite optimal treatment of the underlying disease. Despite wide variation across health service models, we found evidence suggesting an effect on the affective domain of breathlessness, and on psychological outcomes of anxiety and depression. Services were highly valued by patients and carers, who appreciated the education to help them understand their breathlessness, the provision of useful self-management interventions and the provision of expert dignified care which centred on the person. There was however no effect on overall health status or quality of life using varied generic measures, and mixed evidence around any effect on physical function.

To our knowledge, this is the first systematic review in this field; a recent narrative review described some common service features, but focused on an emerging service in Munich that contributed to our data.^49^ The bias of effect towards psychological health outcomes is concordant with the primary focus of these services to support living with breathlessness rather than taking the symptom away. The effect on depression, which in cases arose from preventing its onset within usual care,^21 34^ may have been achieved through expert management of breathlessness and concurrent symptoms, but also through receipt of holistic care that prioritised active listening and putting the person before their disease. The effect sizes observed (point estimates: distress: −0.57, mastery: 0.18, anxiety −0.45, depression: −0.55) are larger than those achieved with psychological therapies, self-management programmes and more comparable to pulmonary rehabilitation,^50 51^ despite the different interventional approach. While few measurable effects were identified for physical function and quality of life, we feel it important to acknowledge the qualitative data that captured participants’ feelings of expanding horizons, including being able to maintain daily activities and tasks. The diverse nature of patient-reported improvements in physical function may be difficult to capture using standardised measures, and individualised measures (eg, goal attainment scaling^52^) could have more utility in this setting.

We intentionally made no attempt to compare with pulmonary rehabilitation, although in chronic respiratory disease the interface between the two service models must be addressed. In no respect do we view holistic breathlessness services as a replacement for pulmonary rehabilitation, which is a highly effective and underused intervention.^14^ These services may however act as a next step for people who remain highly symptomatic despite completing pulmonary rehabilitation, as a bridge for people limited by chronic breathlessness but who decline pulmonary rehabilitation (which may include people who are post-admission for an acute exacerbation), and/or as an adjunct for patients whose goals relate to psychosocial health. Home-based pulmonary rehabilitation provision overcomes some issues with transportation and improves reach.^53 54^ However, holistic breathlessness service services may provide an additional opportunity for health gains in people unable to complete programmes with a major exercise component, particularly where breathlessness limits people from exercising to an intensity associated with a training response.^6 16 55^ The inclusion of palliative care may also be helpful for this population, who have distressing physical and psychological symptoms, often limited understanding of their disease, and infrequently discuss end-of-life issues in routine clinical care.^24^ Although international guidelines advocate for early integration of palliative care in chronic disease,^22 23^ the unpredictable course of many respiratory conditions, including COPD, and the difficulty of predicting survival are barriers to timely palliative care referral and receipt. A symptom-triggered approach should reach more people likely to benefit than current approaches based on prognostication.^24^ For services already well-aligned with palliative care, adoption of the core therapeutic components for breathlessness management into existing practice may suffice.

The heterogeneity of service models with respect to staffing, structure, content and target populations is an important finding. While some shared characteristics were identified (Box 3), further work is required to determine the most effective components, and which recipients gain most benefit. This includes determining optimal service duration, particularly as one trial found better outcomes for distress due to breathlessness and mastery after one session versus three (hypothesising that one session increased self-efficacy and reduced logistic challenges of multiple clinic visits). The literature is small but increasing, and new data from services identified with no published outcomes can be expected. Use of consistent measures may permit meta-regression, or responder analysis using individual level data to identify service and patient characteristics related to better outcomes. An alternative approach is to use discrete choice experiments to identify which components would be prioritised and preferred by patients and carers, particularly in resource-limited settings. Findings would inform future services as appropriate, but also adoption of the most effective characteristics into existing services upstream. Increased consideration of cost effectiveness is also warranted.

Box 3 **Typical composition of a holistic breathlessness service**

Intended users

- People with advanced disease and chronic breathlessness despite optimal disease management, and their informal carers

Philosophy

- Optimising the person’s ability to live with and self-manage breathlessness, with a focus on the person before their disease

Staffing

- Multidisciplinary team of experts in breathlessness and dignified care

Setting

- Mixture of face-to-face support in clinics and/or at home, and phone support

Interventions

- Information and education, psychological support, self-management strategies and other appropriate interventions

Strengths of this work include a registered protocol, and a systematic and comprehensive search across multiple databases, inclusive of grey literature, with no exclusions by publication year or language. Eligibility and quality assessment was conducted independently by two authors, and multiple stakeholders (researchers, clinicians, service user representatives) contributed to the analysis and interpretation of these data. The review also has some limitations. First, the meta-analyses included data from services shown to vary in structure, delivery and recipients. We completed sensitivity analyses in response to any clinical heterogeneity, but the overall dataset was moderate in size and sensitivity analysis compromised the precision of our effect size estimates. These estimates may be inflated by lack of blinding of study personnel in some instances, and disappointment effects in control groups where a fast-track design was not used. Moreover, although we did not assess for statistical evidence of publication bias, there was clear evidence of selective reporting where study authors did not provide data for statistically non-significant findings. Some of our estimates do not include these data, and caution should be applied in these instances. There were also challenges with inconsistent use of, and unclear reporting of, outcome measures, which sometimes precluded meta-analysis (eg, breathlessness intensity). For the qualitative synthesis, included data were drawn from published studies or abstracts. This created an additional layer of abstraction, although also allowed synthesis of study authors’ interpretations as we did not limit data extraction to direct quotations. Qualitative data were predominantly drawn from two UK services,^19–21^ and patients who had fully engaged with the services. Less is understood about experiences of these services internationally, of carers, and of those who dropped out and perhaps might report less benefit. Finally, we limited the review to studies in people with advanced disease, which reflects key studies in the current evidence base. We acknowledge that service access based on disease severity may however not serve those patients with distressing breathlessness but in early stages of disease by traditional markers, for example, moderate airflow obstruction or potentially resectable thoracic cancer. As such, we advocate access to these services primarily based on the presence of breathlessness, accepting the empirical data presented does not extend to some groups.

In conclusion, holistic services for chronic breathlessness in people with advanced disease overall demonstrate positive effects on patient distress due to breathlessness and psychological health. Services are heterogonous in their content and delivery, but are highly valued by patients and families, who appreciate tailored education around breathlessness, provision of simple, portable self-management interventions and expert staff providing person-centred, dignified care. Chronic or distressing breathlessness can serve as an appropriate referral indicator for timely referral and receipt of palliative care, especially in non-cancer conditions where prognostication causes delays. Further work should test and understand the most effective service configurations and how these can be integrated into existing healthcare systems.
