# Supplementary material for: Holistic services for people with advanced disease and chronic breathlessness: a systematic review and meta-analysis
Source: Thorax. 2018 Nov 29;74(3):270–81. doi: 10.1136/thoraxjnl-2018-211589 (PMC6467249; doi:10.1136/thoraxjnl-2018-211589)
Supplement: Supplementary data [file thoraxjnl-2018-211589supp001.pdf]

Holistic services for people with advanced disease and chronic breathlessness: a systematic review and meta-analysis – Supplementary material

Authors: Lisa Jane Brighton[¶], Sophie Miller[¶], Morag Farquhar, Sara Booth, Deokhee Yi, Gao Wei, Sabrina Bajwah, William D-C. Man, Irene J Higginson, Matthew Maddocks

[¶]Joint first authors

APPENDIX 1: MEDLINE search strategy

1 exp Palliative Care/
2 exp Terminal Care/
3 exp Terminally Ill/
4 exp Hospices/
5 ((advanc* or progressiv* or agressiv* or end) adj2 (diagnos* or diseas* or illnes* or cancer* or malignan* or stage* or dementia* or failure* or heart*)).tw.
6 (last adj3 life).tw.
7 (Advanced disease or Cancer or Intrathoracic malignancy or Lung cancer or Chronic obstructive pulmonary disease or Non-malignant disease).tw.
8 1 or 2 or 3 or 4 or 5 or 6 or 7
9 exp nursing/
10 exp physical therapy modalities/
11 exp occupational therapy/
12 (Multiprofessional or Multidisciplinary or Holistic or Complex intervention or Non-pharmacological intervention or non-pharmacological management or non-pharmacological or physiotherap* or Nurs* or Occupational therap*).tw.
13 dyspn?ea.tw.
14 (short* adj2 breath).tw.
15 (urge* adj2 breath*).tw.
16 breathless*.tw.
17 ((labo?red or difficult* or small) adj3 breath*).tw.
18 ((respirat* or breath*) adj3 (distress* or comfort* or discomfort*)).tw.
19 (air adj3 (hunger or starve* or need* or gasp* or pant*)).tw.
20 suffocat*.tw.
21 unsatisf* inspiration.tw.
22 exp Dyspnea/
23 (breathlessness intervention service or breathlessness support service).tw.
24 9 or 10 or 11 or 12
25 13 or 14 or 15 or 16 or 17 or 18 or 19 or 20 or 21 or 22
26 24 and 25
27 23 or 26
28 8 and 27

TABLE S1: Outcomes measured in controlled studies

**Outcomes**	**Studies**												**Total**
	Barton et al. (2010)[1]	Bredin et al. (1999)[2]	Corner et al. (1996)[3]	Farquhar et al. (2014)[4]	Farquhar et al. (2016)[5]	Higginson et al. (2014)[6]	Johnson et al. (2015)[7]	Pearce et al. (2006)[8,a]	Yates et al. (2007) *Quasi-Exp*[9,a]	Yates et al. (2007) *RCT*[9,a]	Yates et al. (2011)[10,a]	Yorke et al. (2015)[11]	
Breathlessness													
Intensity (Best/worst/average)	x	x	x			x	x	x	x	x	x	x	10
Distress	x	x	x	x	x		x		x	x	x	x	10
Mastery				x	x	x	x	x					5
Other Symptoms													
Anxiety/Depression	x	x	x	x	x		x	x			x	x	9
Cough												x	1
Fatigue							x	x				x	3
General		x				x						x	3
Functioning		x	x	x	x	x							5
Quality of life		x		x	x	x						x	5
Service use	x			x	x	x	x						5
Survival		x				x							2
Carer distress/burden				x	x								2

[a] *– abstract only*

TABLE S2: QualSyst Quality assessment – Quantitative

Author	1.Objective	2.Design	3.Subject selection	4.Subject characteristics	5.Random allocation	6. Blinding investigators	7.Blinding subjects	8.Outcomes	9.Sample size	10.Analysis	11.estimate of variance	12.confounding	13.Results	14. conclusion	Total Score
Barton et al. (2010)[1]	Yes	Yes	Yes	Yes	Yes	No	NA	Yes	Yes	Yes	Yes	Yes	Yes	Yes	92%
Bredin et al. (1999)[2]	Yes	Yes	Partial	Yes	Yes	No	NA	Yes	Yes	Partial	Yes	Yes	Yes	Partial	81%
Corner et al. (1996)[3]	Yes	Yes	Yes	Yes	Partial	No	NA	Yes	Yes	Yes	Yes	Yes	Yes	Yes	88%
Farquhar et al. (2014)[4]	Yes	Yes	Yes	Yes	Yes	Yes	NA	Yes	Yes	Yes	Yes	Yes	Yes	Yes	100%
Farquhar et al. (2016)[5]	Yes	Yes	Yes	Yes	Yes	Yes	NA	Yes	Yes	Yes	Yes	Yes	Yes	Yes	100%
Higginson et al. (2014)[6]	Yes	Yes	Yes	Yes	Yes	Yes	NA	Yes	Yes	Yes	Yes	Yes	Yes	Yes	100%
Johnson et al. (2015)[7]	Yes	Yes	Yes	Yes	Yes	No	NA	Yes	Yes	Yes	Yes	Yes	Yes	Yes	92%
Pearce et al. (2006)[8,a]	Partial	Yes	No	No	Partial	No	NA	Yes	Partial	Partial	Yes	Partial	Partial	Yes	54%
Yates et al.(2007)[9,a]															
-Quasi-experimental study	Yes	Yes	No	No	No	No	NA	No	Partial	Partial	No	No	Partial	Yes	35%
-RCT	Yes	Yes	No	No	Partial	No	NA	No	Partial	Partial	No	Yes	Partial	Yes	46%
Yates et al. (2011)[10,a]	Yes	Yes	Partial	Partial	Partial	No	NA	Partial	Yes	Partial	Yes	Yes	Partial	Yes	69%
Yorke et al (2015)[11]	Yes	Yes	Yes	Yes	Yes	No	NA	Yes	Yes	Yes	Yes	Yes	Yes	Yes	92%

[a] *– abstract only*

TABLE S3: Measures of breathlessness intensity in controlled studies:

**Descriptor**	**Scale**	**Timeframe**	**Studies**								
			Barton et al. (2010)[1]	Bredin et al. (1999)[2]	Corner et al. (1996)[3]	Higginson et al. (2014)[6]	Johnson et al. (2015)[7]	Pearce et al. (2006)[8 a]	Yates et al. (2007)[9 a]	Yates et al. (2011)[10 a]	Yorke et al. (2015)[11]
Best	VAS	Past week			X						
		Unknown		X							
	Unknown	Unknown							X	X	
Worst	VAS	Past week			X						
		Unknown		X							
	NRS	Past 24 hours	X			X	X				X
	Unknown	Unknown								X	
Average	NRS	-	X				X				X
		Past 24 hours				X					
	Unknown	Unknown								X	
On exertion	Borg	-						X			
	NRS	Past 24 hours				X					
At Rest	Borg	-						X			
Now	NRS	-	X								

[a] – *abstract only*; NRS – numerical rating scale, VAS – Visual analogue scale

TABLE S4: QualSyst Quality assessment – Qualitative

Author	1. objective	2. design	3. Context	4.Connection to literature	5. Sampling	6. Data collection	7.Analysis	8.credibility	9.Conclusions	10.Reflexivity	Total Score
Booth et al. (2006)[12]	Partial	Yes	Yes	Yes	Yes	Yes	Yes	Yes	Yes	No	84%
Corner et al. (1996)[3]	Yes	Yes	Yes	Yes	Yes	No	No	No	Yes	No	60%
Farquhar et al. (2014)[4]	Yes	Yes	Yes	Partial	Yes	Yes	Yes	Yes	Yes	No	85%
Farquhar et al. (2016)[5]	Yes	Yes	Yes	Yes	Yes	Yes	Yes	No	Yes	No	80%
Farquhar et al. (2010a)[13,a]	Yes	Yes	Partial	Partial	No	Partial	No	No	Partial	No	40%
Farquhar et al. (2010b)[14]	Partial	Partial	Yes	Yes	Partial	No	No	No	Yes	No	45%
Gysels et al. (2015, 2016)[15 16]	Yes	Yes	Yes	Yes	Yes	Yes	Yes	No	Yes	No	80%
Hately et al (2003)[17]	Partial	Yes	Yes	Partial	Partial	Partial	Partial	No	Partial	No	50%
Higginson et al. (2014)[6]	No	Partial	Yes	Partial	Yes	Yes	Yes	Yes	Yes	No	70%
Reilly et al. (2016)[18]	Yes	Partial	Yes	Yes	Yes	Yes	Partial	No	Yes	No	70%
Wood et al. (2013)[19]	Yes	Yes	Yes	Yes	Partial	Yes	Yes	Yes	Yes	No	85%

[a] – *abstract only*

References

1. Barton R, English A, Nabb S, et al. A randomised trial of high vs low intensity training in breathing techniques for breathless patients with malignant lung disease: a feasibility study. *Lung cancer (amsterdam, netherlands)* 2010;70(3):313-19.
2. Bredin M, Corner J, Krishnasamy M. Multicentre randomised controlled trial of nursing intervention for breathlessness in patients with lung cancer. *BMJ (Clinical Research Edition)* 1999;318(7188):901-04.
3. Corner J, Plant H, Ahern R, et al. Non-pharmacological intervention for breathlessness in lung cancer. *Palliative Medicine* 1996;10(4):299-305.
4. Farquhar MC, Prevost AT, McCrone P, et al. Is a specialist breathlessness service more effective and cost-effective for patients with advanced cancer and their carers than standard care? Findings of a mixed-method randomised controlled trial. *BMC medicine* 2014;12:194.
5. Farquhar MC, Prevost AT, McCrone P, et al. The clinical and cost effectiveness of a Breathlessness Intervention Service for patients with advanced non-malignant disease and their informal carers: mixed findings of a mixed method randomised controlled trial. *Trials* 2016;17:185.
6. Higginson IJ, Bausewein C, Reilly CC, et al. An integrated palliative and respiratory care service for patients with advanced disease and refractory breathlessness: a randomised controlled trial. *The Lancet Respiratory medicine* 2014;2(12):979-87.
7. Johnson MJ, Kanaan M, Richardson G, et al. A randomised controlled trial of three or one breathing technique training sessions for breathlessness in people with malignant lung disease. *BMC medicine* 2015;13:213.
8. Pearce L, MacLeod V, Baker A, et al. Randomised controlled trial of nurse-led breathlessness intervention to improve the management of breathlessness in patients with chronic obstructive pulmonary disease at a district general hospital. *Thorax* 2006;61:II84-II84.
9. Yates P, White E, Skerman H. Evaluating alternate approaches for delivering non-pharmacological interventions for dyspnea in patients with lung cancer...Oncology Nursing Society 32nd Annual Congress, April 24-27, 2007, Las Vegas, NV. *Oncology Nursing Forum* 2007;34(2):551-51.
10. Yates P, Hardy J, Kwun F, et al. A randomised controlled trial of a nonpharmacological intervention for dyspnoea. *Asia-Pacific Journal of Clinical Oncology* 2011;7:82.
11. Yorke J, Lloyd-Williams M, Smith J, et al. Management of the respiratory distress symptom cluster in lung cancer: a randomised controlled feasibility trial. *Supportive Care in Cancer* 2015;23(11):3373-84.
12. Booth S, Farquhar M, Gysels M, et al. The impact of a breathlessness intervention service (BIS) on the lives of patients with intractable dyspnea: A qualitative phase 1 study. *Palliative & Supportive Care* 2006;4(3):287-93.
13. Farquhar M, Higginson IJ, Booth S. Modelling the carer support component of a complex intervention for breathlessness in advanced disease. *Palliative Medicine* 2010;24(4 SUPPL. 1):S167-S68.
14. Farquhar M, Higginson IJ, Fagan P, et al. Results of a pilot investigation into a complex intervention for breathlessness in advanced chronic obstructive pulmonary disease (COPD): brief report. *Palliative & Supportive Care* 2010;8(2):143-49.
15. Gysels M, Reilly CC, Jolley CJ, et al. How does a new breathlessness support service affect patients? *The European respiratory journal* 2015;46(5):1515-8.
16. Gysels M, Reilly CC, Jolley CJ, et al. Dignity Through Integrated Symptom Management: Lessons From the Breathlessness Support Service. *Journal of Pain and Symptom Management* 2016;52(4):515-24.
17. Hately J, Laurence V, Scott A, et al. Breathlessness clinics within specialist palliative care settings can improve the quality of life and functional capacity of patients with lung cancer. *Palliative Medicine* 2003;17(4):410-17.
18. Reilly CC, Bausewein C, Pannell C, et al. Patients' experiences of a new integrated breathlessness support service for patients with refractory breathlessness: Results of a postal survey. *Palliative Medicine* 2016;30(3):313-22.
19. Wood H, Connors S, Dogan S, et al. Individual experiences and impacts of a physiotherapist-led, non-pharmacological breathlessness programme for patients with intrathoracic malignancy: A qualitative study. *Palliative Medicine* 2013;27(6):499-507.
